# Ischemic stroke of undetermined source as a priming event for brain metastasis: a case report and systematic literature review

**DOI:** 10.3389/fonc.2025.1638420

**Published:** 2025-08-20

**Authors:** Paolo Amisano, Antonio Ciacciarelli, Svetlana Lorenzano, Irene Berto, Sara Belcastro, Danilo Toni, Manuela De Michele

**Affiliations:** ^1^ Department of Human Neurosciences, Sapienza University of Rome, Rome, Italy; ^2^ Stroke Unit, Emergency Department, Umberto I Hospital, Sapienza University of Rome, Rome, Italy; ^3^ Department of Radiological Sciences, Oncology and Pathology, I.C.O.T. Hospital, Sapienza University of Rome, Latina, Italy

**Keywords:** embolic stroke of undetermined source (ESUS), neoplastic embolism, cancer-associated coagulopathy, cancer-related stroke, brain metastases (BMS), pre-metastatic niche

## Abstract

The association between ischemic stroke (IS) and malignancy is well established. Cancer-related strokes are predominantly embolic and classified as embolic strokes of undetermined source (ESUS). While malignancy-associated coagulopathy represents the primary pathogenic mechanism, neoplastic embolization of circulating tumor cells is another potential etiology, particularly in cases of cardiac and pulmonary malignancies. We report the case of a 58-year-old man who presented with ESUS and concurrent multiple pulmonary nodules. Despite a comprehensive oncologic evaluation, including an ultrasound-guided bronchoscopic biopsy of the suspected lesion, no malignancy was detected. Several months later, the patient developed focal seizures, and brain magnetic resonance imaging (MRI) revealed multiple left frontoparietal space-occupying lesions with imaging features suggestive of brain metastases (BMs). A follow-up whole-body computed tomography (CT) scan confirmed a right upper lobe lung mass, which was diagnosed as pulmonary adenocarcinoma on subsequent mediastinal lymph node biopsy. The ischemic event may have contributed to the subsequent development of BMs through neoplastic embolization, allowing malignant cells to proliferate within the ischemic brain parenchyma. Alternatively, the stroke may have resulted from cancer-associated coagulopathy. In this context, post-stroke pathophysiological changes—including blood–brain barrier (BBB) disruption, neuroinflammation, hypoxia-induced angiogenesis, and extracellular matrix (ECM) remodeling—may have created a permissive microenvironment for tumor cell seeding and colonization, definable as a “pre-metastatic niche”. ESUS can be the initial clinical manifestation of an undiagnosed malignancy, and IS itself may facilitate tumor dissemination, ultimately leading to BMs. This case underscores the importance not only of a thorough oncologic workup in patients with ESUS, but also of a strict neuroradiological follow-up, as a delayed diagnosis of cancer-related stroke may allow malignancy progression, significantly worsening the prognosis and limiting therapeutic options.

## 1 Introduction

The association between ischemic stroke (IS) and malignancy is well recognized, with approximately 4% to 10% of individuals presenting with IS having a concurrent cancer diagnosis (1). In oncologic patients, stroke is associated with increased morbidity and higher short-term mortality, further complicating disease management and overall prognosis (2).

While these conditions share common risk factors such as aging, smoking, obesity, and alcohol consumption, there are multiple cancer-specific pathophysiological mechanisms that may lead to cerebrovascular events (3).

Malignancy-related strokes are predominantly classified as embolic stroke of undetermined source (ESUS) (4). Indeed, the primary mechanism underlying IS in cancer is malignancy-associated coagulopathy, characterized by hyperactivation of the coagulation cascade, platelet dysfunction, endothelial injury, and tumor-induced inflammation (3); these patients often exhibit elevated levels of D-dimer, thrombin–antithrombin complexes, tissue factor, endothelial adhesion molecules, and antiphospholipid antibodies, all contributing to a prothrombotic state and an increased risk of thromboembolic events (5). Stroke in this context can also result from non-bacterial thrombotic endocarditis (NBTE), paradoxical embolization from a deep vein thrombosis in the presence of a patent foramen ovale (PFO), or, less commonly, *in situ* thrombus formation within the cerebral circulation; the latter is typically observed in severe disseminated intravascular coagulation (6).

Beyond these hypercoagulability-driven mechanisms, neoplastic embolism—i.e., the fragmentation and embolization of a portion of a solid tumor—represents a distinct etiology of cancer-related stroke. This phenomenon is primarily associated with intracardiac tumors, pulmonary malignancies invading the pulmonary veins or left atrium, or paradoxical embolism via right-to-left cardiac shunting (7).

This case report describes a patient with an initial diagnosis of ESUS, who subsequently developed brain metastasis (BM) from pulmonary adenocarcinoma in the same cerebral region.

Although the hypothesis that cancer-associated stroke may promote BMs has been occasionally proposed in the literature, it remains a poorly understood and infrequently documented phenomenon.

Through this case study and a comprehensive systematic literature review, we provide an in-depth analysis of this association, focusing on the potential pathophysiological mechanisms linking IS and the spread of BMs. Understanding this interplay is crucial, as IS significantly worsens the prognosis of oncologic patients (8).

Indeed, IS can add neurological disability to an already fragile patient population, thereby compromising functional status and quality of life. Moreover, IS often requires a temporary suspension or modification of anticancer therapies. Finally, according to the proposed hypothesis, IS itself could promote metastatic dissemination, further aggravating the overall disease course.

## 2 Case report

A 58-year-old man with a medical history of smoking, arterial hypertension, type II diabetes mellitus, and chronic ischemic heart disease treated with triple coronary artery bypass surgery in 2019 presented to the emergency department with a 24-h history of aphasia. His home medications included aspirin, a proton pump inhibitor, diuretics, antihypertensive agents, and insulin. Neurological examination revealed expressive aphasia [National Institutes of Health Stroke Scale (NIHSS) 2], prompting an angio-computed tomography (CT) scan of the intra- and extracranial vessels, which showed localized areas of cortical–subcortical hypodensity in the left frontotemporal region, consistent with subacute ischemic lesions, in the absence of large vessel occlusions. Additionally, at the lower field of view, an incidental finding of multiple solid nodular formations with irregular margins was noted in the upper lobes of both lungs, the largest located in the right lung and measuring approximately 2 cm.

**Figure 1:**
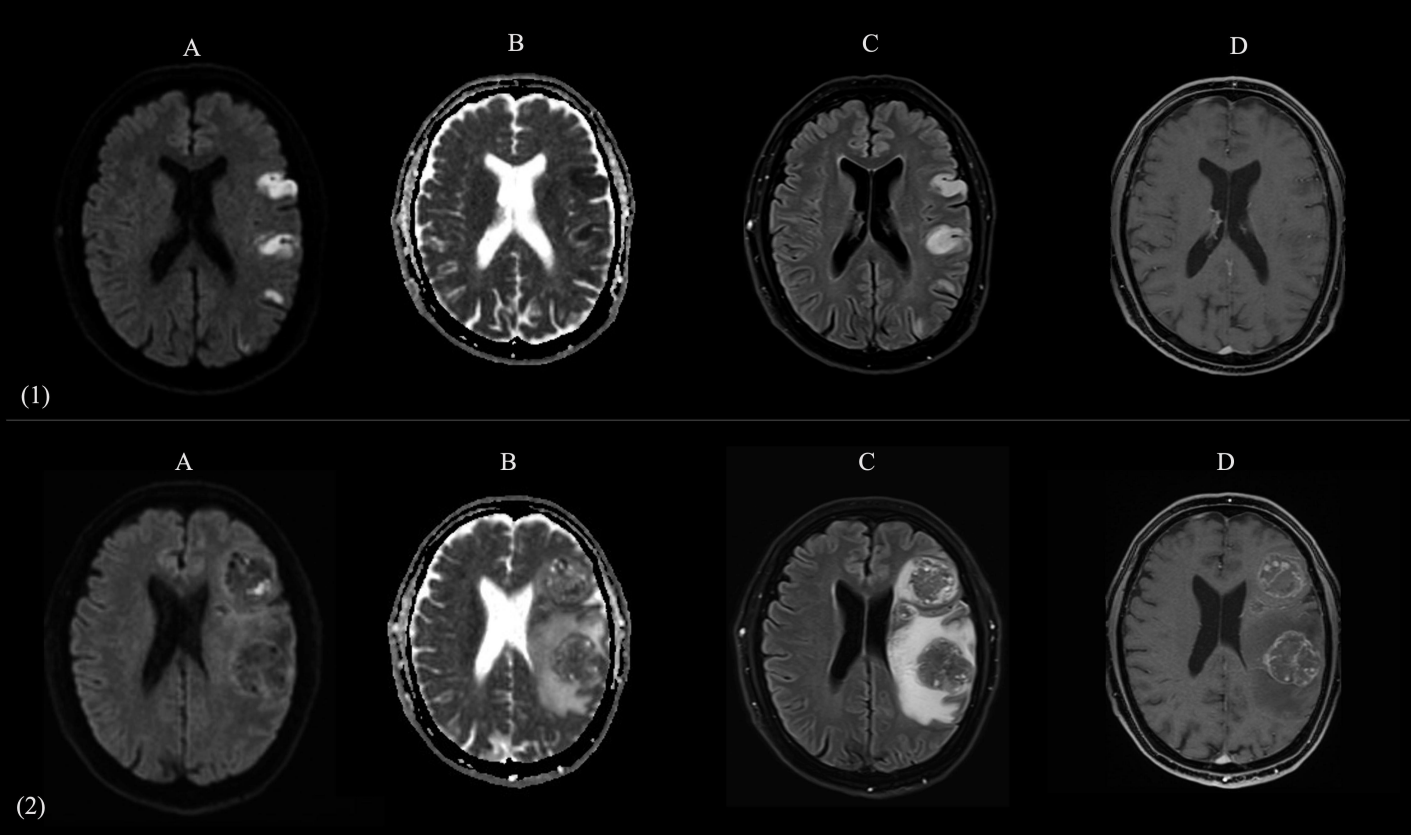
Comparative brain MRI at Time 0 (acute presentation of stroke) and Time 1 (onset of seizure). Two-panel composite figure (panels 1 and 2, images A–D) illustrating the evolution of left parietal lobe lesions in the same patient. The panel 1 (1A–1D) shows axial MR images acquired at Time 0 (emergency department presentation for acute onset of aphasia and right hemiparesis). The panel 2 (2A–2D) presents the corresponding sequences obtained at Time 1 (several months later, during evaluation for new-onset epileptic seizures). **(A)**: Diffusion-weighted imaging (DWI) shows multiple cortical-subcortical hyperintensities in the left parietal lobe at Time 0, consistent with acute ischemic infarcts; at Time 1, these regions are replaced by expansile lesions with mass effect. **(B)**: Apparent diffusion coefficient (ADC) maps confirm restricted diffusion in the same areas at Time 0, consistent with acute ischemia; at Time 1, the lesions show heterogeneous ADC signal, with regions of reduced ADC consistent with solid, highly cellular tumor components, and regions of increased ADC reflecting necrotic changes and surrounding vasogenic edema. **(C)**: FLAIR images reveal initial hyperintense ischemic lesions at Time 0, compatible with established infarcts; at Time 1, large mixed-intensity expansile lesions are present in the same regions, surrounded by marked vasogenic edema. **(D)**: Post-contrast T1-weighted imaging at Time 1 demonstrates multiple ring-enhancing masses in the same distribution as the prior ischemic lesions, consistent with brain metastases.

**Figure 2:**
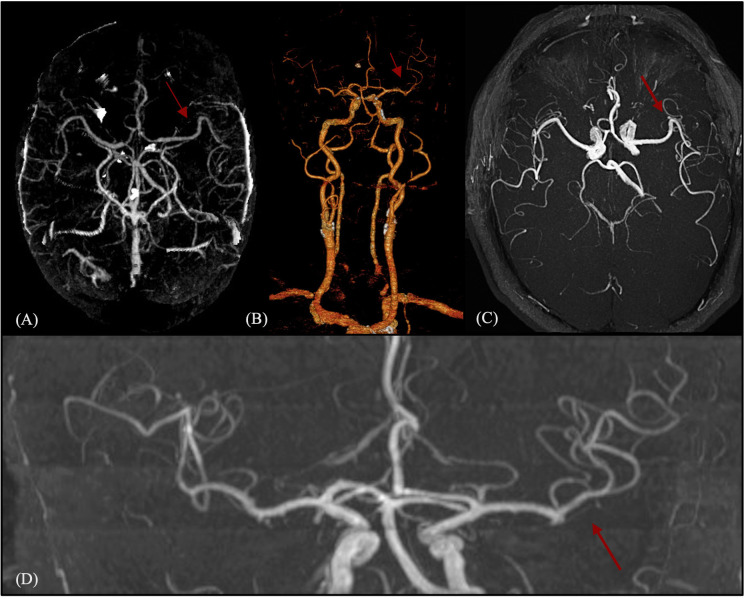
Acute vascular imaging (CT angiography and MR angiography) at stroke onset. One-panel composite figure (images A–D) showing the intracranial vascular status in the acute phase. Neuroimaging demonstrates the absence of large vessel occlusion or pathological anomalies of the circle of Willis. The red arrows indicate a normally patent middle cerebral artery without proximal or distal occlusion. **(A)** Multiplanar maximum intensity projection (MIP) reconstruction from triphasic CT angiography. **(B)** 3D volume-rendered reconstruction from triphasic CT angiography. **(C, D)** Post-processed images from high-resolution time-of-flight (TOF) MR angiography: axial view (C) and coronal view (D).

The patient was admitted to our Stroke Unit, where a brain magnetic resonance imaging (MRI) with angiography confirmed multiple early subacute ischemic lesions in the territory of the distal branches of the left middle cerebral artery (**Figure 1**, panel 1), with no evidence of large vessel occlusion or vascular abnormalities (**Figure 2**). As part of the workup to determine stroke etiology, comprehensive blood tests and autoimmune and infectious screenings were performed, revealing solely elevated glycated hemoglobin levels (8.2%). Transthoracic echocardiography (TTE) was unremarkable, with preserved global systolic function and no atrial enlargement. Transesophageal echocardiography (TEE) ruled out potential cardiac embolic sources, showing no evidence of PFO, atrial septal aneurysm, or intracardiac thrombi. Carotid and transcranial Doppler ultrasounds revealed mild carotid atherosclerosis, with predominantly fibrocalcific plaques, no features of instability, and no hemodynamically significant stenosis.

**Figure 3:**
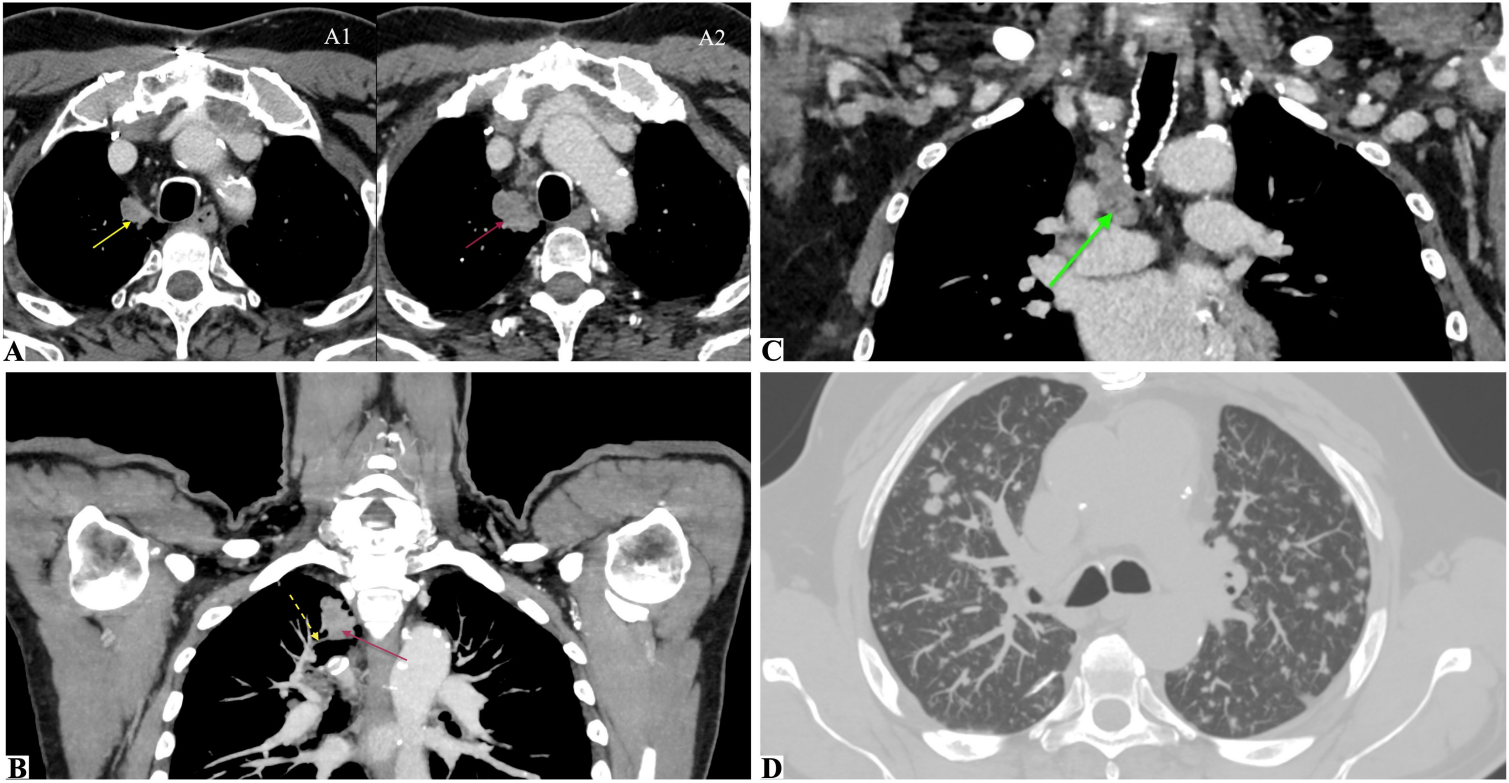
CT imaging at Time 0 (initial detection of a suspicious pulmonary nodule) and Time 1 (confirmed adenocarcinoma with metastatic spread). Multimodal chest CT assessment illustrating the evolution of a right upper lobe pulmonary nodule. **(A1, A2)** Axial CT scans at Time 0 (A1) show a paramediastinal pulmonary nodule in the apical segment of the right upper lobe (yellow arrow), with clear dimensional increase on follow-up CT at Time 1, 5 months later (A2, magenta arrow). **(B)** Maximum intensity projection (MIP) reconstruction at Time 1 displaying the same nodule (magenta arrow) with suspected pathological invasion of a branch of the right superior pulmonary vein (dashed yellow arrow). **(C)** CT scan at Time 1 showing an enlarged right paratracheal lymph node (green arrow), which was sampled during histological staging. **(D)** CT with MIP reconstruction at Time 1 showing multiple secondary pulmonary nodules diffusely distributed across both lungs, consistent with metastatic dissemination.

To further investigate the suspected pulmonary lesions, a whole-body CT scan was performed, confirming the presence of multiple solid pulmonary nodules bilaterally, the largest measuring 18 mm on the right, with irregular margins and early cavitation (**Figure 3A**). Diffuse gastric wall thickening was also noted, leading to gastroscopy, which was unremarkable. Repeated serial bronchoalveolar lavages with cytological and microbiological examinations were negative for neoplastic cells. In addition, an ultrasound-guided bronchoscopic biopsy was performed on the largest paratracheal nodule in the right upper pulmonary lobe, which was clearly visualized on imaging. Histological examination of the biopsy sample did not reveal malignant cells; however, the specimen was moderately paucicellular and consisted predominantly of fibrino-hematic material with scattered inflammatory components. The patient was discharged with a diagnosis of ESUS on dual antiplatelet therapy and statins, with a referral for loop recorder implantation to monitor cardiac activity, as well as a recommended chest CT and follow-up pulmonary consultation at 3 months.

Five months later, the patient returned to the emergency department with episodic speech disturbances, confusion, and burning paresthesia in the right upper limb, which were interpreted as focal seizures. Notably, the recommended chest imaging and pulmonary evaluation had not yet been performed at that time. A brain MRI with angiography revealed five space-occupying lesions in the left frontoparietal region, ranging in size between 0.5 and 4.2 cm. Contrast-enhanced imaging revealed features suggestive of neoangiogenesis, including irregular, disorganized perilesional vessels and intense peripheral enhancement, surrounding a central non-enhancing necrotic core. Gradient-recalled echo (GRE) showed hypointensities indicative of hemorrhage. Surrounding edema caused mass effect on the left lateral ventricle and a mild rightward midline shift (**Figure 1**, panel 2). Given the radiological findings consistent with BMs, steroid therapy and mannitol therapy were initiated. Levetiracetam 500 mg twice daily was started.

A control whole-body CT scan revealed a parenchymal mass in the apical segment of the right upper lung lobe (3.5 × 2 cm) with central necrosis, infiltrating the mediastinal pleura, with associated multiple necrotic lymphadenopathies in the right paratracheal (2.5 cm), hilar, and subcarinal regions, also with suspected pathological invasion of a branch of the right superior pulmonary vein. Multiple secondary-appearing pulmonary nodules (**Figure 3**) and a left adrenal gland lesion consistent with metastasis were also observed. An ultrasound-guided biopsy was performed on a thoracic lymph node located in the right lower paratracheal region, which revealed a solid-pattern adenocarcinoma with PD-L1 expression <1% by immunohistochemistry, absence of ALK rearrangement, and no actionable mutations in EGFR, KRAS, BRAF, or ROS1, as determined by targeted next-generation sequencing. The patient subsequently received cranial radiotherapy for BMs and systemic chemoimmunotherapy, in line with current treatment guidelines for advanced non-small cell lung cancer (NSCLC) without targetable molecular alterations.

## 3 Systematic literature review

### 3.1 Methods

We conducted a systematic review of the literature using the PubMed database, without language restrictions, to identify reports relevant to the hypothesis of IS as a potential facilitator or permissive environment for metastatic seeding.

The search covered all articles published between 1 January 1950 and 7 July 2025, with the following search strategy:

((“Stroke”[MeSH Terms] OR “Ischemic Stroke”[MeSH Terms] OR stroke[tiab] OR ischemic stroke[tiab]) AND (cancer[tiab] OR malignancy[tiab] OR neoplasm[tiab] OR neoplasms[MeSH Terms]) AND (neoplastic embolization[tiab] OR neoplastic embolisation[tiab] OR neoplastic embolism[tiab] OR metastatic niche[tiab] OR tumor embolism[tiab] OR cancer-associated embolism[tiab] OR tumor embolisation[tiab] OR tumor embolization[tiab] OR tumor microenvironment[tiab] OR tumor niche[tiab] OR pre-metastatic niche[tiab] OR neoplastic microenvironment[tiab])).

Titles and abstracts were independently screened by two reviewers (PA and AC). A total of 45 articles were excluded due to lack of relevance based on topic or scope. A total of 43 articles were retained for full-text review.

Additionally, five further studies were identified through manual screening of citations and references in relevant reviews and articles. Among the 48 total records deemed pertinent, 6 were selected for final inclusion: 5 clinical case reports and 1 retrospective cohort study.

All studies reporting IS preceding BMs were considered eligible, especially when addressing a possible temporal or pathophysiological link, irrespective of study type.

This review was conducted in accordance with the PRISMA (Preferred Reporting Items for Systematic Reviews and Meta-Analyses) guidelines.

**Figure 4:**
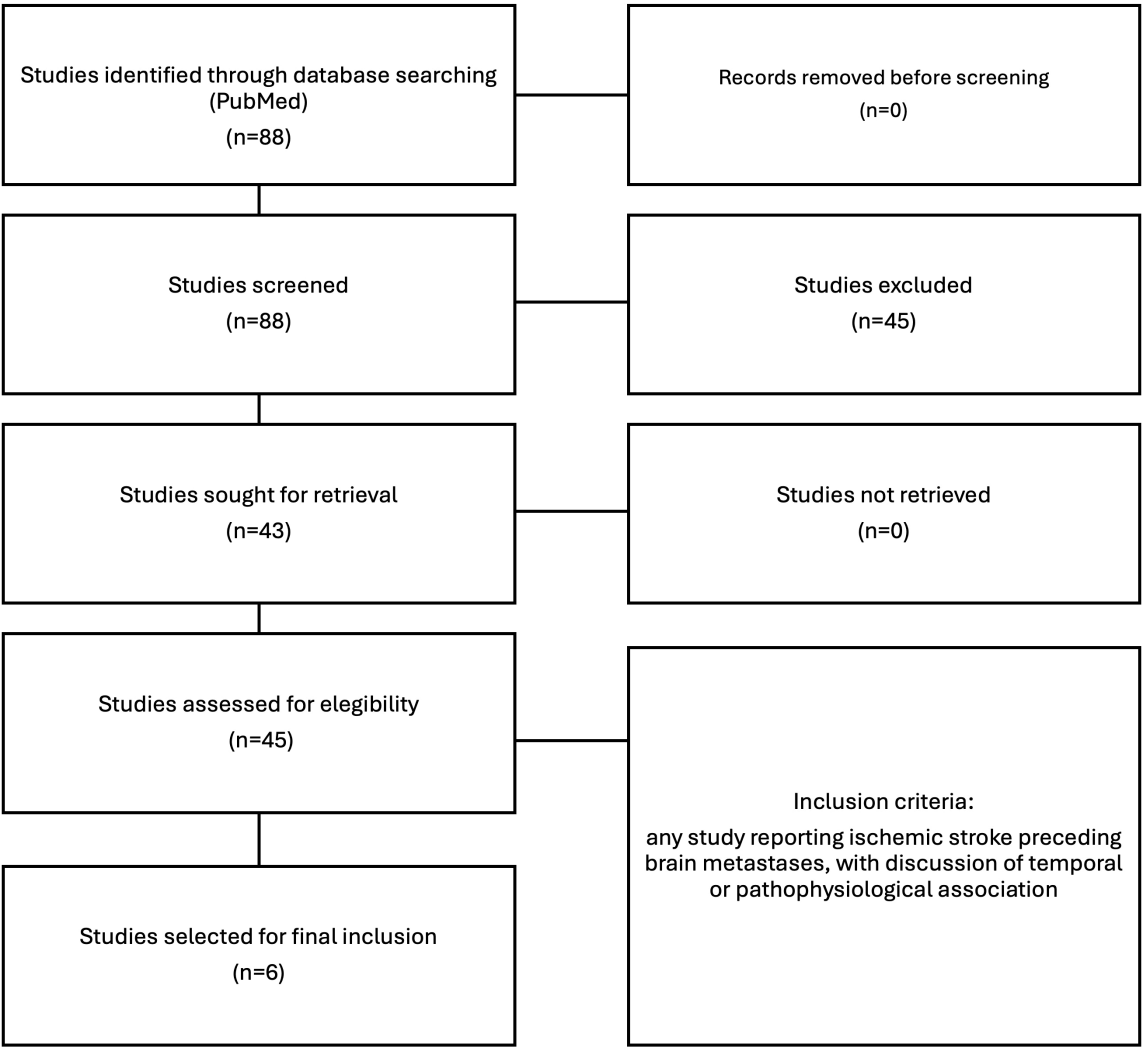
PRISMA flow diagram of study selection.

A flow diagram illustrating the selection process is provided in **Figure 4**.

### 3.2 Review result

Over the past decades, only a limited number of studies have explored the potential association between IS and subsequent BMs, the majority of which are case reports.

As summarized in **Table 1**, the first case was described by Nielsen and Posner in 1983 (9), involving a woman with cervical carcinoma. Other authors described similar findings in patients with diverse primary malignancies, including cardiac sarcoma (10), carotid intimal sarcoma (11), renal cell carcinoma (12), and malignant melanoma (13). In all these reports, BMs developed within the same vascular territory as the prior ischemic event, sometimes after a long latency period, and were generally associated with poor outcomes.

**Table 1:** Summary of published cases and studies reporting the occurrence of brain metastases (BMs) at the site of prior cerebral infarction.

Authors and year	Type of study	Age/Sex	Primary cancer	Infarct location	Metastasis location	Time from stroke to BM	Outcome
Nielsen & Posner, 1983 (9)	Case report	50/F	Cervical carcinoma	Left MCA territory	Site of prior infarction	4 weeks	Death within 2 months of discharge
Sun et al., 2017 (10)	Case report	33/M	Cardiac sarcoma	Right hemisphere	Site of prior infarction	4 months	Alive at 1 year from BM diagnosis
Agarwal et al., 2020 (11)	Case report	52/M	Carotid intimal sarcoma	Bilateral (left > right) infarcts	Site of prior infarction	4 weeks	On palliative care at the time of writing the report
Gwak et al., 2021 (12)	Case report	48/M	Renal cell carcinoma	Right MCA and PCA territory	Site of prior infarction	8 months	Death within 6 months of discharge
Atallah et al., 2023 (13)	Case report	22/F	Malignant melanoma	Left MCA & ACA territory	Site of prior infarction	16 years	Death within 8 months of discharge
Kim et al., 2020 (7)	Retrospective observational study on 307 patients with NSCLC, 52 with BM	Mean age 64/185 M, 122 F	Non-small cell lung cancer	Variable	Site of prior infarction	Not assessed	Worse overall survival in patients with BM with prior IS vs. patients with BM without IS
Present case	Case report	58/M	Pulmonary adenocarcinoma	Left MCA territory	Site of prior infarction	5 months	Alive at 6 months from BM diagnosis

Kim et al. (7) (**Table 1**) retrospectively analyzed 307 patients diagnosed with NSCLC to investigate the relationship between IS and BMs. Among these cases, 52 patients had developed BMs, and the prevalence of prior IS was specifically assessed and compared between those with and without BMs. The study found that IS was significantly more frequent in patients with NSCLC who subsequently developed BMs compared to those who did not (84.6% vs. 62.7%, *p* = 0.002). Moreover, multivariate analysis confirmed prior IS as an independent risk factor for the development of BMs.

## 4 Discussion

Neoplastic embolism, resulting from the intravascular migration of solid tumor components, is a rare and often underrecognized mechanism of ESUS in oncologic patients, accounting for approximately 5% of cancer-related strokes (14). This phenomenon is primarily associated with intracardiac tumors and solid malignancies involving heart or the pulmonary veins (15–23). Left atrial or aortic valve-adjacent tumors pose the highest threat (24, 25), particularly left atrial myxomas, which have a 45% embolic risk (26). Tumor embolization has also been observed in invasive head and neck cancers (14).

The embolic process can occur following direct vascular invasion and tumor mass fragmentation, which most often results from intra-tumoral hemorrhage. Tumor emboli consist of both malignant cells and thrombotic material, reflecting the typical cancer-related hypercoagulable state (27).

In pulmonary malignancies, tumor cells may intravasate into the venous circulation and form emboli that reach the brain, most often via a pulmonary arteriovenous shunt—possibly induced by the tumor itself—or via a PFO, thereby bypassing pulmonary capillary filtration (4). In some cases, however, direct infiltration of the pulmonary veins may serve as a route for cerebral embolization.

In strokes caused by tumor embolism, it has been hypothesized that, if patients survive long enough, these emboli may serve as a nidus for malignant cell proliferation within the brain parenchyma, ultimately leading to the onset of BMs (14, 28, 29).

However, this phenomenon remains poorly described in the literature and mostly speculative.

In our case, IS may have resulted from direct tumor embolization, with subsequent BM development through this very mechanism.

The presence of pulmonary nodules—initially biopsied with negative results but subsequently confirmed as adenocarcinoma—suggests a false-negative finding, supporting the hypothesis of embolism from an occult malignancy. Notably, chest CT performed in the venous phase at the onset of BMs revealed a suspected invasion of a branch of the right superior pulmonary vein, indicating possible tumor cell intravasation—an abnormality not clearly visible on the initial scan. Moreover, TEE did not reveal an intracardiac right-to-left shunt or pathological pulmonary arteriovenous fistulas. Histopathological analysis of a thrombus retrieved through mechanical thrombectomy could have provided stronger support for this hypothesis; however, such analysis was not feasible, as the patient did not present with a large vessel occlusion.

This limitation weakens our ability to definitively confirm neoplastic embolism as the cause of stroke in this case.

An alternative pathophysiological mechanism to consider in our case is that IS resulted from cancer-associated coagulopathy.

This scenario would be consistent with a paraneoplastic stroke caused by a malignancy-induced prothrombotic state, even before the primary tumor was diagnosed.

Cancer-associated coagulopathy is a well-documented paraneoplastic manifestation that can precede malignancy detection. Research indicates that up to 20% of patients with cryptogenic IS may harbor an occult malignancy (30–32).

In this context, IS may have acted as a priming event for the development of BMs by creating a permissive microenvironment that favored the seeding and progression of circulating tumor cells. This reflects the “seed and soil” paradigm of metastatic spread, whereby circulating tumor cells (“seeds”) colonize receptive tissues (“soil”)—in this case, the post-ischemic brain (33).

The pre-metastatic niche is a well-established concept describing microenvironmental modifications that render distant organs permissive to tumor colonization even before the arrival of circulating neoplastic cells (34–36). This process is orchestrated by primary tumor-derived factors, bone marrow-derived cells, and local stromal alterations (37).

Chronic inflammation plays a crucial role, as pro-inflammatory mediators—such as S100A8/A9, tumor necrosis factor-α (TNF-α), and the TLR4-NF-κB signaling pathway—recruit myeloid cells and activate the endothelium, facilitating tumor extravasation (38). Vascular remodeling, driven by tumor-secreted pro-angiogenic factors like vascular endothelial growth factor (VEGF) and angiopoietin-2, induces the formation of abnormal, highly permeable blood vessels that enhance the dissemination and survival of tumor cells (39, 40). The lymphatic network expansion, stimulated by VEGF-C and VEGF-D, further facilitates tumor spread (41), while local immunosuppression, mediated by myeloid-derived suppressor cells (MDSCs), tumor-associated macrophages, and regulatory T cells (Tregs), dampens anti-tumor immune responses (42, 43). Concurrently, metabolic reprogramming, including shifts in glucose metabolism and lipid utilization, sustains metastatic cell growth (44), while extracellular matrix remodeling, driven by fibronectin and matrix metalloproteinases (MMPs), provides structural support for tumor invasion (45).

The IS microenvironment undergoes strikingly similar changes.

A key feature of ischemic injury is blood–brain barrier (BBB) disruption, which increases vascular permeability and facilitates the extravasation of circulating cells and soluble factors, a process reminiscent of endothelial dysfunction in metastatic progression (46). Hypoxia-driven neovascularization leads to the formation of immature, fragile vessels that, while aiming to restore perfusion, also create a permissive environment for tumor cell survival and invasion (47). The post-stroke inflammatory response further reinforces this effect, as activated microglia, astrocytes, and infiltrating immune cells release cytokines—such as TNF-α, interleukin-1β (IL-1β), and S100 proteins—which are also implicated in pre-metastatic niche formation (48). The recruitment of bone marrow-derived myeloid cells contributes to tissue remodeling and angiogenesis (49), while lymphangiogenesis provides an additional route for immune and tumor cell trafficking (50). Moreover, ischemic tissue adapts to hypoxia by shifting towards glycolysis and altered lipid metabolism, a metabolic phenotype that closely resembles the tumor microenvironment (51). Simultaneously, extracellular matrix remodeling, characterized by increased fibronectin and MMP activity, enhances cellular adhesion and migration, supporting both tissue repair and potential tumor engraftment (52). The emerging immunosuppressive landscape in the subacute and chronic phases of stroke—marked by an expansion of Tregs, MDSCs, and increased TGF-β and IL-10 production—mirrors the immune evasion mechanisms seen in metastatic progression (53).

Taken together, these findings suggest that cerebral infarction can trigger a cascade of molecular and cellular changes that closely resemble those involved in metastatic priming, transforming the ischemic brain into a pre-metastatic niche, leading to the development of BMs.

Recent experimental findings provide strong biological support for the proposed hypothesis. In a preclinical study published in *Frontiers in Neuroscience* in 2019, Prakash et al. (54) demonstrated that a prior IS significantly promotes the development of BMs in a murine model. Mice subjected to transient middle cerebral artery occlusion, followed by intracardiac injection of melanoma cells, developed a substantially greater metastatic burden in the ischemic hemisphere compared to non-stroke controls, with metastases preferentially localizing to peri-infarct regions.

Histological and molecular analyses revealed profound post-ischemic alterations, including angiogenic remodeling, increased endothelial permeability, and a sustained pro-inflammatory environment characterized by the upregulation of adhesion and chemotactic molecules. These changes collectively shaped a permissive neurovascular niche that facilitated tumor cell adhesion, extravasation, and colonization.

This hypothesis offers another plausible explanation for IS and the subsequent onset of BMs in our patient. Its plausibility is further supported by the occurrence of a synchronous systemic tumor dissemination, as observed in the whole-body follow-up CT, which involved other organs such as the adrenal gland.

## 5 Limitations

In our case, the proposed pathophysiological link between IS and BMs remains speculative, owing to the absence of direct biological data. Specifically, no thrombotic material was available for histopathological examination, and no peri-infarct tissue could be sampled.

Additionally, the current body of evidence on this relationship remains limited and largely anecdotal, as most published reports are isolated case descriptions lacking systematic biological characterization. Even the study by Kim et al. (7), despite its larger sample size and structured analysis, is retrospective in nature and therefore insufficient to establish a causal link.

Nonetheless, our hypotheses are biologically and pathophysiologically highly plausible, as strongly supported by preclinical research. The available literature—although limited—tends to converge in this direction.

Crucially, well-designed prospective human studies are needed to confirm this association and clarify its underlying mechanisms. Future research should focus on *in vivo* investigations, ideally combining advanced neuroimaging, biomarker profiling, and histopathological analysis of thrombi retrieved from thrombectomy procedures, as well as of peri-infarct brain tissue.

## 6 Conclusion

This case highlights a potential association between IS and BMs, raising the hypothesis that cerebral infarction may act as a pre-metastatic niche facilitating tumor cell seeding.

Moreover, the diagnostic challenge posed by cancer-related ESUS underlines the need for a structured oncological evaluation and close neuroradiological follow-up in patients with ESUS, to support timely cancer detection and tailored clinical management.

## Data Availability

The original contributions presented in the study are included in the article/supplementary material. Further inquiries can be directed to the corresponding author.

## References

[1] Lun R Siegal DM . Cancer-associated ischemic stroke: current knowledge and future directions. Bleed Thromb Vasc Biol. (2024). 3(s1). doi: 10.4081/btvb.2024.117

[2] Zuber M . Stroke and cancer. Rev Neurol (Paris). (2023) 179:417–24. doi: 10.1016/j.neurol.2023.03.009, PMID: 37024364

[3] Sun MY Bhaskar SMM . When two maladies meet: disease burden and pathophysiology of stroke in cancer. Int J Mol Sci. (2022) 23:15769. doi: 10.3390/ijms232415769, PMID: 36555410 PMC9779017

[4] Heo JH Yun J Kim KH Jung JW Yoo J Kim YD . Cancer-associated stroke: thrombosis mechanism, diagnosis, outcome, and therapeutic strategies. J Stroke. (2024) 26:164–78. doi: 10.5853/jos.2023.03279, PMID: 38836266 PMC11164583

[5] Erritzøe-Jervild M Wenstrup J Hougaard BH Kruuse C . Diagnosing cancer-associated ischemic stroke: a systematic review of hematological biomarkers. Int J Stroke. (2024) 19:622–34. doi: 10.1177/17474930241227385, PMID: 38192106

[6] Dardiotis E Aloizou AM Markoula S Siokas V Tsarouhas K Tzanakakis G . Cancer-associated stroke: pathophysiology, detection and management (Review). Int J Oncol. (2019) 54:779–96. doi: 10.3892/ijo.2019.4669, PMID: 30628661 PMC6365034

[7] Kim J Jung G Kim HG Kim JY Yang GY Kim YZ . Role of cancer emboli as a metastatic core on the growth of brain metastasis in patients with non-small cell lung cancer. J Neurointens Care. (2020) 3:12–9. doi: 10.32587/jnic.2020.00227

[8] Seystahl K Hug A Weber SJ Kapitza S Gramatzki D Wanner M . Cancer is associated with inferior outcome in patients with ischemic stroke. J Neurol. (2021) 268:4190–202. doi: 10.1007/s00415-02110528-3, PMID: 33945004 PMC8505392

[9] Nielsen SL Posner JB . Brain metastasis localized to an area of infarction. J Neurooncol. (1983) 1:191–5. doi: 10.1007/BF00165602, PMID: 6206208

[10] Sun YP Wang X Gao YS Zhao S Bai Y . Primary cardiac sarcoma complicated with cerebral infarction and brain metastasis: A case report and literature review. Cancer biomark. (2017) 21:247–50. doi: 10.3233/CBM-170448, PMID: 29060931 PMC13075743

[11] Agarwal S Derman A Raz E Hoda ST Arcot K Yaghi S . Carotid intimal sarcoma causing stroke and intracranial metastasis via tumor embolization. Neurology. (2020) 94:e1122–5. doi: 10.1212/WNL.0000000000008980, PMID: 31949089

[12] Gwak DS Hwang YH Kim YW . Case report: brain metastasis confined to the infarcted area following stroke. Front Neurol. (2021) 11:617142. doi: 10.3389/fneur.2020.617142, PMID: 33584517 PMC7878549

[13] Atallah O Chaurasia B . Brain metastasis localized to the same area of infarction: illustrative case. J Neurosurg Case Lessons. (2023) 6:CASE23325. doi: 10.3171/CASE23325, PMID: 37581584 PMC10555590

[14] Baker R Bakali Z Crocker JS Mowla A Smith M Grossman A . Tumor embolic stroke: the importance of pathological assessment of clots after thrombectomy. J Clin Med. (2024) 13:1834. doi: 10.3390/jcm13071834, PMID: 38610599 PMC11012646

[15] Hussain SMA . Tumor embolism and acute arterial occlusion: A systematic review. Surg Open Sci. (2022) 10:216–22. doi: 10.1016/j.sopen.2022.10.006, PMID: 36389271 PMC9664516

[16] Imaizumi K Murate T Ohno J Shimokata K . Cerebral infarction due to a spontaneous tumor embolus from lung cancer. Respiration. (1995) 62:155–6. doi: 10.1159/000196412, PMID: 7569337

[17] Navi BB Kawaguchi K Hriljac I Lavi E DeAngelis LM Jamieson DG . Multifocal stroke from tumor emboli. Arch Neurol. (2009) 66:1174–5. doi: 10.1001/archneurol.2009.172, PMID: 19752313

[18] Grazziotin MU Turnipseed WD . Arterial tumor embolism caused by metastatic melanoma: case report and literature review. J Vasc Surg. (2002) 36:191–3. doi: 10.1067/mva.2002.123327, PMID: 12096280

[19] Gómez JR Vañó J Luengo L Escuder J Castellote M Ros S . Tumor embolism after pneumonectomy for primary pulmonary neoplasia. Ann Vasc Surg. (1995) 9:199–203. doi: 10.1007/BF02139664, PMID: 7786706

[20] Dimitrović A Breitenfeld T Supanc V Roje-Bedeković M Butković Soldo S Vargek-Solter V . Stroke caused by lung cancer invading the left atrium. J Stroke Cerebrovasc Dis. (2016) 25:e66–8. doi: 10.1016/j.jstrokecerebrovasdis.2015.12.043, PMID: 26922131

[21] Oyama T Asai T Miyazawa T Yokoyama K Kogure Y Torii A . A case of cerebral tumor embolism from extracardiac lung cancer treated by mechanical thrombectomy. NMC Case Rep J. (2020) 7:101–5. doi: 10.2176/nmccrj.cr.2019-0205, PMID: 32695556 PMC7363642

[22] Takasugi J Sakaguchi M Oyama N Gon Y Terasaki Y Sasaki T . Recurrent stroke due to metastatic pulmonary tumor emboli as an important clinical entity. J Stroke Cerebrovasc Dis. (2017) 26:e108–10. doi: 10.1016/j.jstrokecerebrovasdis.2017.03.012, PMID: 28366663

[23] Nakanishi K Kawano H Yamagishi Y Kamma H Shiokawa Y Hirano T . Tumor cells detected in retrieved thrombus: cancer-associated stroke. Intern Med. (2021) 60:2491–4. doi: 10.2169/internalmedicine.6201-20, PMID: 33678737 PMC8381188

[24] ElBardissi AW Dearani JA Daly RC Mullany CJ Orszulak TA Puga FJ . Embolic potential of cardiac tumors and outcome after resection: a case-control study. Stroke. (2009) 40:156–62. doi: 10.1161/STROKEAHA.108.525709, PMID: 18948602

[25] Singh A Jenkins DP Dahdal M Dhar S Ratnatunga CP . Recurrent arterial embolization from a metastatic germ cell tumor invading the left atrium. Ann Thorac Surg. (2000) 70:2155–6. doi: 10.1016/s0003-4975(00)01899-3, PMID: 11156144

[26] Wold LE Lie JT . Cardiac myxomas: a clinicopathologic profile. Am J Pathol. (1980) 101:219–40., PMID: 7446701 PMC1903582

[27] Bloch RS Jacobs LA Lewis LS Bernys CF . Malignant tumor embolism: a rare presentation of Malignant disease. J Cardiovasc Surg. (1986) 27:630–1., PMID: 3760030

[28] Navi BB Kasner SE Elkind MSV Cushman M Bang OY DeAngelis LM . Cancer and embolic stroke of undetermined source. Stroke. (2021) 52:1121–30. doi: 10.1161/STROKEAHA.120.032002, PMID: 33504187 PMC7902455

[29] O’Neill BP Dinapoli RP Okazaki H . Cerebral infarction as a result of tumor emboli. Cancer. (1987) 60:90–5. doi: 10.1002/1097-0142(19870701)60:1<90::aid-cncr2820600116>3.0.co;2-c, PMID: 3581035

[30] Tieck MP Single C Poli S Kowarik MC Ziemann U Mengel A . Screening tools for Malignancy in patients with cryptogenic stroke: systematic review. Eur Stroke J. (2025) 26:23969873241310760. doi: 10.1177/23969873241310760, PMID: 40008556 PMC11866335

[31] Navi BB Zhang C Miller BR Pawar A Cushman M Kasner SE . Diagnosis of incident cancer after cryptogenic stroke: an exploratory analysis of the ARCADIA randomized trial. Neurology. (2024) 103:e210027. doi: 10.1212/WNL.0000000000210027, PMID: 39481070 PMC11527484

[32] Ntaios G Baumgartner H Doehner W Donal E Edvardsen T Healey JS . Embolic strokes of undetermined source: a clinical consensus statement of the ESC Council on Stroke, the European Association of Cardiovascular Imaging and the European Heart Rhythm Association of the ESC. Eur Heart J. (2024) 45:1701–15. doi: 10.1093/eurheartj/ehae150, PMID: 38685132 PMC11107123

[33] Liu Q Zhang H Jiang X Qian C Liu Z Luo D . Factors involved in cancer metastasis: a better understanding to ‘seed and soil’ hypothesis. Mol Cancer. (2017) 16:176. doi: 10.1186/s12943-017-0742-4, PMID: 29197379 PMC5712107

[34] Liu Y Cao X . Characteristics and significance of the pre-metastatic niche. Cancer Cell. (2016) 30:668–81. doi: 10.1016/j.ccell.2016.09.011, PMID: 27846389

[35] Kaplan RN Riba RD Zacharoulis S Bramley AH Vincent L Costa C . VEGFR1-positive haematopoietic bone marrow progenitors initiate the pre-metastatic niche. Nature. (2005) 438:820–7. doi: 10.1038/nature04186, PMID: 16341007 PMC2945882

[36] Sleeman JP . The metastatic niche and stromal progression. Cancer Metastasis Rev. (2012) 31:429–40. doi: 10.1007/s10555-012-9373-9, PMID: 22699312 PMC3470821

[37] Hiratsuka S Watanabe A Aburatani H Maru Y . Tumour-mediated upregulation of chemoattractants and recruitment of myeloid cells predetermines lung metastasis. Nat Cell Biol. (2008) 10:1039–45. doi: 10.1038/ncb1507, PMID: 17128264

[38] Yang J Li M Zheng QC . Emerging role of Toll-like receptor 4 in hepatocellular carcinoma. J Hepatocell Carcinoma. (2015) 2:11–7. doi: 10.2147/JHC.S44515, PMID: 27508190 PMC4918281

[39] Peinado H Alečković M Lavotshkin S Matei I Costa-Silva B Moreno-Bueno G . Melanoma exosomes educate bone marrow progenitor cells toward a pro-metastatic phenotype through MET. Nat Med. (2016) 22:1502. doi: 10.1038/nm1216-1502b, PMID: 27923027

[40] Huang Y Song N Ding Y Yuan S Li X Cai H . Pulmonary vascular destabilization in the premetastatic phase facilitates lung metastasis. Cancer Res. (2009) 69:7529–37. doi: 10.1158/0008-5472.CAN-08-4382, PMID: 19773447

[41] Wakisaka N Hasegawa Y Yoshida S Yamada A Ugumori T Wang Q . Primary tumor-secreted VEGF-A increases lymphatic vessel density in pre-metastatic lymph nodes and promotes distant metastasis. Cancer Res. (2015) 75:3408–17. doi: 10.1371/journal.pone.0144056, PMID: 26630663 PMC4668078

[42] Olkhanud PB Damdinsuren B Bodogai M Gress RE Sen R Wejksza K . Tumor-evoked regulatory B cells promote breast cancer metastasis by converting resting CD4+ T cells to T-regulatory cells. Cancer Res. (2011) 71:3505–15. doi: 10.1158/0008-5472.CAN-10-4316, PMID: 21444674 PMC3096701

[43] Sceneay J Chow MT Chen A Halse HM Wong CS Andrews DM . Primary tumor hypoxia recruits CD11b+/Ly6Cmed/Ly6G+ immune suppressor cells and induces chemoresistance through a nitric oxide-dependent mechanism. Cancer Res. (2012) 72:5025–34. doi: 10.1158/0008-5472.CAN-11-3873, PMID: 22751463

[44] Fong MY Zhou W Liu L Alontaga AY Chandra M Ashby J . Breast-cancer-secreted miR-122 reprograms glucose metabolism in pre-metastatic niche to promote metastasis. Nat Cell Biol. (2015) 17:183–94. doi: 10.1038/ncb3094, PMID: 25621950 PMC4380143

[45] Zhang H Yu Y Zhou L Ma J Tang K Xu P . Circulating tumor microparticles promote lung metastasis by reprogramming inflammatory and mechanical niches via a macrophage-dependent pathway. Cancer Immunol Res. (2018) 6:1046–56. doi: 10.1158/2326-6066.CIR-17-0574, PMID: 30002156

[46] Yang Y Rosenberg GA . Blood-brain barrier breakdown in acute and chronic cerebrovascular disease. Stroke. (2011) 42:3323–8. doi: 10.1161/STROKEAHA.110.608257, PMID: 21940972 PMC3584169

[47] Marti HJ Bernaudin M Bellail A Schoch H Euler M Petit E . Hypoxia-induced vascular endothelial growth factor expression precedes neovascularization after cerebral ischemia. Am J Pathol. (2000) 156:965–76. doi: 10.1016/S0002-9440(10)64964-4, PMID: 10702412 PMC1876841

[48] Lambertsen KL Biber K Finsen B . Inflammatory cytokines in experimental and human stroke. J Cereb Blood Flow Metab. (2012) 32:1677–98. doi: 10.1038/jcbfm.2012.88, PMID: 22739623 PMC3434626

[49] Jin R Yang G Li G . Inflammatory mechanisms in ischemic stroke: role of inflammatory cells. J Leukoc Biol. (2010) 87:779–89. doi: 10.1189/jlb.1109766, PMID: 20130219 PMC2858674

[50] Esposito E Ahn BJ Shi J Nakamura Y Park JH Mandeville ET . Brain-to-cervical lymph node signaling after stroke. Nat Commun. (2019) 10:5306. doi: 10.1038/s41467-019-13324-w, PMID: 31757960 PMC6876639

[51] Hu X De Silva TM Chen J Faraci FM . Cerebral vascular disease and neurovascular injury in ischemic stroke. Circ Res. (2017) 120:449–71. doi: 10.1161/CIRCRESAHA.116.308427, PMID: 28154097 PMC5313039

[52] Rempe RG Hartz AM Bauer B . Matrix metalloproteinases in the brain and blood-brain barrier: versatile breakers and makers. J Cereb Blood Flow Metab. (2016) 36:1481–507. doi: 10.1177/0271678X16655551, PMID: 27323783 PMC5012524

[53] Liesz A Suri-Payer E Veltkamp C Doerr H Sommer C Rivest S . Regulatory T cells are key cerebroprotective immunomodulators in acute experimental stroke. Nat Med. (2009) 15:192–9. doi: 10.1038/nm.1927, PMID: 19169263

[54] Prakash R Izraely S Thareja NS Lee RH Rappaport M Kawaguchi R . Regeneration enhances metastasis: A novel role for neurovascular signaling in promoting melanoma brain metastasis. Front Neurosci. (2019) 13:297. doi: 10.3389/fnins.2019.00297, PMID: 31024232 PMC6465799

